# Importance of Inhibitor Surveillance During Emicizumab Prophylaxis in Young Children With Hemophilia: An Illustrative Case Series

**DOI:** 10.1155/crh/5572714

**Published:** 2025-11-10

**Authors:** Kelly A. Bush, Marc Durocher, Jacqueline Limjoco, Courtney D. Thornburg

**Affiliations:** ^1^Hemophilia and Thrombosis Treatment Center, Rady Children's Hospital San Diego, San Diego, California, USA; ^2^Department of Pediatrics, University of California Health Sciences, La Jolla, California, USA

**Keywords:** babies, clotting factor concentrate, emicizumab, hemophilia, prophylaxis

## Abstract

Hemophilia is an X-linked inherited bleeding disorder associated with bleeding, which starts in infancy. The age of initiation of prophylaxis with clotting factor concentrate is limited by the intravenous mode of administration. Emicizumab, a Factor VIII (FVIII) mimetic, may be initiated for prophylaxis in persons with hemophilia A (HA) in infancy, given the subcutaneous route of administration. Bleeds that occur while on emicizumab prophylaxis are treated with clotting factor concentrate. The primary risk of clotting factor concentrate is inhibitor development, with the highest risk occurring within the first 10–20 exposure days. Individuals on emicizumab who develop inhibitors may still use emicizumab for prophylaxis but require a change in bleed management. We report bleeding and inhibitor outcomes in six infants with severe HA, who were effectively treated with emicizumab prophylaxis starting at a median age of 8 months old. Two cases were diagnosed with high-titer inhibitors during surveillance testing performed after initiation of emicizumab. They continued emicizumab for prophylaxis and changed bleed management to Recombinant factor VIIa (rFVIIa). This report highlights the importance of ongoing inhibitor surveillance during emicizumab prophylaxis to ensure inhibitor detection, which requires a change in bleed management.

## 1. Introduction

Hemophilia A (HA) is an X-linked recessive bleeding disorder caused by a deficiency of Factor VIII (FVIII). The current standard of care for treatment of severe HA is prophylaxis with FVIII clotting factor concentrate (CFC) or FVIII mimetic therapy [1]. The median age of prophylaxis initiation for severe HA has decreased over time to 13.1 months (IQR: 10.4–19.1) [2]; however, limitations include the need for reliable venous access, if CFC is used for prophylaxis, and risk of inhibitor development at any time during CFC exposure, with the highest risk occurring within the first 10–20 factor exposure days (EDs) [3]. Emicizumab (Roche), a subcutaneously administered FVIII mimetic with a half-life of 4–5 weeks, is approved for prophylaxis without age restriction for persons with HA (PwHA) with inhibitor (PwHAi) (2017) and for PwHA without inhibitors (2018) [4–6]. CFC or bypassing therapy (for PwHAi) is still required for bleed, injury, and surgical management.

Initial clinical trials were focused on previously treated patients with FVIII inhibitors who had more than 50 EDs. Until recently, data on the efficacy and safety of emicizumab in minimally treated patients (MTPs) (≤ 5 factor EDs) and previously untreated patients (PUPs), as well as previously treated patients with or without inhibitors less than 24 months of age, were limited to case reports and case series [7–14]. In 2021, HAVEN 7, a Phase IIIb, multicenter, open-label, single-arm study to evaluate the efficacy, safety, pharmacokinetics, and pharmacodynamics of subcutaneous emicizumab in patients from birth to 12 months of age with HA without inhibitors (NCT04431726), was initiated to evaluate emicizumab in PUPs and MTPs without FVIII inhibitors, and the primary analysis results were published in December 2023 [15]. Prior to the initiation of HAVEN 7, we conducted a retrospective study with the primary objective to describe real-world clinical experience on the initiation of emicizumab prophylaxis in infants and toddlers.

## 2. Methods

### 2.1. Study Population

The retrospective study included six children less than 24 months of age with severe HA who were prescribed emicizumab prophylaxis and managed at Rady Children's Hospital San Diego (RCHSD) Hemophilia and Thrombosis Treatment Center (HTTC) between November 2017 and April 2020. The institutional review board at UC San Diego approved the research protocol with waiver of consent and Health Insurance Portability and Accountability Act (HIPAA) authorization for retrospective review of clinical data available between November 2017 and April 2020. No written consent has been obtained from the patients as there is no patient identifiable information included in this case series. The patients included in this case series did not participate in the HAVEN 7 clinical trial, and all patients who met the study inclusion criteria were included.

Given the retrospective nature, medical treatment and laboratory testing were not informed by the study.

In clinical practice at our HTC, emicizumab dosing frequency is informed by discussion with the parents and available vial sizes. Parents selected weekly dosing if they felt it would improve their injection skills or improve adherence. Parents selected every 2-week dosing if they wanted to continue the same dose with the maintenance regimen as with the loading regimen or wanted a balance between less frequent dosing and lower dose volume. Parents selected every 4-week dosing if they favored the least injections per month and/or if the dosing regimen utilized the fewest number of vials (least wastage) per 4 weeks of dosing. FVIII inhibitor testing is guided by the National Bleeding Disorders Foundation (NBDF) Medical and Scientific Advisory Council Document #236 recommendations on standardized testing and surveillance for inhibitors for patients with HA and B in response to CFC exposure, switch in CFC, planned surgical procedures, and lack of clinical or laboratory response to CFC (Table 1) [16]. In clinical practice, however, testing is sometimes deferred for patients without a history of inhibitor, particularly in the absence of clinical suspicion and/or CFC exposure. Inhibitor testing with a bovine chromogenic assay is required for accurate results in the presence of emicizumab. At RCHSD, HTTC testing is completed through the Centers for Disease Control and Prevention Community Counts Registry [17] or Versiti Blood Research Institute, Diagnostic Laboratories.

### 2.2. Data Collection

Data were abstracted from the electronic health record for demographics, hemophilia diagnosis, inhibitor history, bleeding and treatment history, surgical history, reason for initiating emicizumab, age, weight, dosing regimen for emicizumab treatment, and adverse reactions.

## 3. Results

### 3.1. Case Series

This report includes six patients between 1 and 23 months of age. Clinical case descriptions are provided in the following and in Table 2. In summary, the median age at initiation of prophylaxis was 8 months (range: 1–23), and the median FVIII EDs prior to emicizumab initiation were 5.5 (range: 0–15). The most common reason for choosing emicizumab over FVIII prophylaxis was parental preference for the subcutaneous route of administration. One patient had a high-titer FVIII inhibitor prior to initiation. In total, there were 63 patient months of follow-up after initiation of emicizumab (median 9 months, range: 3–24). Notably, two patients had high-titer FVIII inhibitors detected on surveillance testing after initiation of emicizumab. Both continued on emicizumab for prophylaxis and changed bleed management to Recombinant factor VIIa (rFVIIa). There were no reported thromboembolic events or other complications attributed to emicizumab. Antidrug antibody testing was not completed due to a lack of a commercially available assay, and activated thromboplastic time (aPTT) was used as a surrogate in one child with higher-than-expected bleeding (see Case 2).

#### 3.1.1. Case 1

Case 1 is a non-Hispanic, African American male diagnosed with severe HA on day of life two based on family history and hematomas from venipuncture. At the time of diagnosis, emicizumab was not available, and the family declined prophylaxis with CFC due to the burden of venous access and family history of central line complications. Bleeding episodes were treated on demand with standard half-life (SHL) rFVIII replacement. He had seven EDs prior to presenting with a high-titer FVIII inhibitor (27 BU) in the setting of traumatic oral bleeding unresponsive to a dose of rFVIII replacement (ED 8). After discussion with the family, prophylaxis with emicizumab was promptly initiated without concomitant immune tolerance induction (ITI) at 23 months of age. The bleed management plan included rFVIIa via peripheral vein infusions and antifibrinolytics. He had 24 months of follow-up on emicizumab during which he had zero treated bleeds and no thromboembolic events.

#### 3.1.2. Case 2

Case 2 is a Hispanic, White male diagnosed with severe HA at 13 months of age after evaluation of a left scrotal hematoma, requiring surgical drainage. He was treated with extended half-life (EHL) rFVIIIFc replacement for the management of the scrotal hematoma with negative inhibitor testing up to ED 8. On the last day of CFC for bleed management (ED 12), emicizumab was initiated for prophylaxis after discussion of prophylaxis options. Surveillance inhibitor testing following the intensive CFC exposure was performed two weeks after emicizumab initiation and identified a high-titer FVIII inhibitor via bovine chromogenic assay (53 CBU). After inhibitor detection, he continued emicizumab prophylaxis without concomitant ITI and bleed management changed to rFVIIa via peripheral infusion and antifibrinolytics. He had 15 months of follow-up with reported strict medication adherence during which he had 10 soft tissue bleeds, nine with documented trauma (each treated with one dose of rFVIIa) and one suspected spontaneous knee hemarthrosis requiring a 2-day hospital admission for evaluation and management. He also had mucosal bleeding treated with antifibrinolytics. Due to a higher-than-expected frequency of bleeding, aPTT was performed as a screen for a clinically significant antidrug antibody, which resulted in the normal range as expected with emicizumab laboratory effect on plasma-based assays. aPTT was felt to be an effective screen for clinically significant antidrug antibodies, as the presence of clinically significant antiemicizumab antibodies has been shown to return aPTT values to an elevated state, which is in line with what would be an expected value for a PwHA [7–14]. Bleeding decreased over time, and there were no thromboembolic events.

#### 3.1.3. Case 3

Case 3 is a Non-Hispanic, White male diagnosed with severe HA at 4 weeks of life due to family history. He had one ED with EHL rFVIIIFc for accidental head injury at 6 months of life prior to starting prophylaxis with emicizumab at 10 months. He had 11 months of follow-up postemicizumab initiation with a total of four EDs with EHL rFVIIIFc, one for traumatic oral bleeding, one for accidental head injury, and two for peri-operative management of circumcision (6 weeks after emicizumab initiation). A high-titer FVIII inhibitor (21.2 CBU) was identified upon inhibitor surveillance after a total of 5 ED and 9 months after emicizumab initiation. Emicizumab was continued without concomitant ITI and bleed management changed to rFVIIa and antifibrinolytics. He had no treated bleeds in the 2 months of follow-up after inhibitor diagnosis and no thromboembolic events.

#### 3.1.4. Case 4

Case 4 is a non-Hispanic male from Saipan born at 26 weeks of gestation and diagnosed with severe HA based on family history. His neonatal course was complicated by Grade 1 intraventricular hemorrhage and one traumatic soft tissue bleed treated with plasma-derived von Willebrand factor/FVIII concentrate (the CFC product available at the treating hospital in Saipan) for two ED. Prophylaxis with emicizumab was planned at 9 months of age, but at 6 months of age, he presented with a subdural hemorrhage, requiring transfer to our center for neurosurgical and hematologic expertise. His case was previously reported to describe the initiation of emicizumab after treatment of the subdural hemorrhage [9]. Inhibitor testing was negative prior to emicizumab initiation at ED 15. He had 7 months of follow-up during which he had zero treated bleeds.

#### 3.1.5. Cases 5 and 6

Cases 5 and 6 are non-Hispanic, Asian identical twin males born at 37 1/7 weeks. Both twins had bleeding in the newborn period. Case 5 had persistent bleeding from intramuscular injection sites treated with fresh frozen plasma (FFP) and cryoprecipitate prior to the diagnosis of severe HA. Case 5 also had three ED of SHL rFVIII after the diagnosis of severe HA was established for treatment of a soft tissue bleed in the arm attributed to venipuncture. Case 6 had subgaleal hemorrhage and bleeding from intramuscular injection sites, which were also treated with FFP and cryoprecipitate prior to the diagnosis of severe HA. Emicizumab prophylaxis was initiated at 1 month of age in both infants. They had 3 months of follow-up postemicizumab initiation during which both patients had zero treated bleeds. During this time, Case 6 received one ED of SHL rFVIII for symptoms of possible intracranial hemorrhage (ICH); head imaging was normal. No inhibitor testing was completed.

## 4. Discussion

Emicizumab prophylaxis is approved without age restriction for PwHAi and PwHA. We report six cases of emicizumab initiation in infants and toddlers less than 24 months of age, including two with inhibitors detected on surveillance testing after initiation of emicizumab, which highlights the importance of inhibitor surveillance. There were no major adverse reactions, and overall bleeding rates were low, other than the initial treatment phase of one child with a high-titer inhibitor who experienced multiple soft tissue bleeds. rFVIIa was selected for bleed management for those with inhibitors based on clinical guidelines [18] due to the higher risk of thromboembolic events associated with activated prothrombin complex concentrates. There were no thromboembolic events. Our results are comparable to other case reports/case series and the primary analysis of HAVEN 7 [7–14].

Prophylaxis is the standard of care for children with severe HA, and data demonstrate that earlier initiation of FVIII prophylaxis is associated with better joint outcomes [19]. MASAC document 268 suggests early initiation of emicizumab to reduce the risk of ICH, as the highest risk of ICH is within the first 2 years of life, median age 4 months [18, 20]. In our series, the median age of initiating emicizumab prophylaxis was 8 months, with very early initiation at 4 weeks for twins with early bleeding complications. Of note, in the three patients with a family history of hemophilia, there was no delivery or testing plan, and they had delayed diagnosis and bleeding complications that may have been preventable. Increasing recognition of carrier status and risk to offspring could allow for optimal prenatal planning and earlier hemophilia diagnosis and discussion of treatment options.

This case series also includes two patients who had inhibitors identified on surveillance testing with bovine chromogenic inhibitor assays after the initiation of emicizumab prophylaxis. The inhibitor in Case 2 was identified 2 weeks following the initiation of emicizumab on planned surveillance testing due to recent intensive CFC exposure for bleed management. Prior inhibitor testing was negative 3 days prior to starting emicizumab; however, the patient continued to receive additional doses of EHL rFVIIIFc for bleed management up until the day he started emicizumab prophylaxis. Case 2 highlights the importance of inhibitor testing prior to starting emicizumab, as ideally follow-up inhibitor testing would have been sent just prior to starting emicizumab prophylaxis. Both cases also highlight the importance of continued, long-term, inhibitor surveillance after initiation of emicizumab, an inhibitor that develops in response to FVIII exposure, just prior to starting emicizumab or while on emicizumab, which could otherwise go unrecognized until the time of poor response to FVIII for a surgical procedure or major bleed. Given that prophylaxis with emicizumab is expected to decrease the number of bleeds and the rate of FVIII exposure [21], emicizumab may indirectly reduce or delay inhibitor development; however, this has not yet been proven with the data available to date. Testing for inhibitors with a bovine chromogenic assay is required for accurate results in the presence of emicizumab. Outcomes with emicizumab for PwHAi are excellent but require a change in treatment plan to rFVIIa for the management of bleeds and consideration of ITI. Further work is indicated to evaluate the impact of emicizumab on the incidence and timing of FVIII inhibitor development. In addition, ITI using the Atlanta protocol [22], which combines emicizumab and FVIII replacement, is one option to eradicate an inhibitor while continuing emicizumab prophylaxis. Questions remain, however, on whether there is a risk of inhibitor recurrence if patients continue emicizumab alone post-ITI [23, 24]. This is being investigated further in the Preventing Inhibitor Recurrence Indefinitely (PRIORITY) study (NCT04621916).

One toddler with a high-titer inhibitor had higher bleeding rates than expected. aPTT was sent on one occasion in this patient to ensure that it was shortened as expected with emicizumab. Measuring aPTT may be used as an initial screen for clinically significant antiemicizumab antibodies, and it may be helpful to follow the aPTT trend over time in patients where there is clinical concern for antiemicizumab antibodies. However, aPTT will still be shortened from baseline until the emicizumab drug level is very low. In these cases, emicizumab-specific laboratory testing may be warranted for further evaluation [23, 24].

Notably, none of the patients in the series required central venous catheter placement, which avoided potentially life-threatening device complications but resulted in the need for factor administration by healthcare personnel due to a lack of peripheral vein infusion skills. Although bleeding rates on emicizumab are low, training on self-infusion is still important for the management of bleeding. Clinical decision-making discussions about prophylaxis initiation should consider these factors.

Efficacy and safety conclusions from the HAVEN 7 study, small cohort studies, and the cases described here add to the existing literature regarding the feasibility of initiating and outcomes of emicizumab prophylaxis in infants and toddlers with severe HA. We recommend shared decision-making when discussing prophylaxis options with parents, including the time of initiation and product of choice [8, 25]. Future studies may inform the impact on rates of inhibitor development, ICH, joint health, and quality of life. We recommend that all PUPs and MTPs be enrolled in a longitudinal follow-up study such as ATHN Transcends (NCT04398628).

## Figures and Tables

**Table 1:** Inhibitor surveillance recommendations from the National Bleeding Disorders Foundation Medical and Scientific Advisory Council Document 236—Recommendations on Standardized Testing and Surveillance for Inhibitors in Patients with Hemophilia A and B [16].

Indications for inhibitor testing
Before elective invasive procedures
Suboptimal clinical response to clotting factor concentrate
Suboptimal laboratory response to clotting factor concentrate
Before and after switching clotting factor concentrate products
2-3 Weeks after ≥ 5 exposure days
2-3 Weeks after surgery
At least every third exposure day or every 3 months (whichever occurs sooner until 20 exposure days)
Every 3–6 months between 20 and 150 exposure days
After 150 exposure days at least annually

**Table 2:** Clinical and treatment characteristics.

Patient	Upon initiating emicizumab			During emicizumab prophylaxis						
	Age (mo)	Weight (kg)	Prior FVIII ED (*n*)	Frequency of maintenance dosing	Total treated bleeds (*n*)	Traumatic treated bleeds (*n*)	Joint bleed (*n*)	Time to inhibitor diagnosis after initiation (mo)	FVIII ED prior to inhibitor development (*n*)^∗^	Follow-up (mo)
*High-titer FVIII inhibitor identified preinitiation of emicizumab*										
Case 1	23	10.3	8	Weekly, then every 28 d	0	0	0	—	7	24

*High-titer FVIII inhibitor identified postinitiation of emicizumab*										
Case 2	13	11.8	12	Weekly	11	9	1^†^	1	12	15
Case 3 (MTP)	10	9.6	1	Weekly	0	0	0	9	5	11

*No history of inhibitor prior to initiation of emicizumab*										
Case 4	6	9.3	15	Every 28 d	0	0	0	—	N/A	7
Case 5 (MTP)	1	4.4	3	Weekly	0	0	0	—	N/A	3
Case 6 (PUP)	1	6.3	0	Weekly	0	0	0	—	N/A	3

Abbreviations: ED = exposure days; FVIII = Factor VIII; MTP = minimally treated patient; N/A = not applicable; PUP = previously untreated patient.

^∗^FFP and cryoprecipitate are not classified as ED.

^†^Suspected, spontaneous.

## Data Availability

The data that support the findings of this study are available from the corresponding author upon reasonable request.
